# Semi-enzymatic acceleration of oxidative protein folding by *N*-methylated heteroaromatic thiols†

**DOI:** 10.1039/d3sc01540h

**Published:** 2023-06-16

**Authors:** Shunsuke Okada, Yosuke Matsumoto, Rikana Takahashi, Kenta Arai, Shingo Kanemura, Masaki Okumura, Takahiro Muraoka

**Affiliations:** a Department of Applied Chemistry, Graduate School of Engineering, Tokyo University of Agriculture and Technology 2-24-16 Naka-cho Koganei Tokyo 184-8588 Japan muraoka@go.tuat.ac.jp; b Department of Chemistry, School of Science, Tokai University Kitakaname Hiratsuka-shi Kanagawa 259-1292 Japan; c Institute of Advanced Biosciences, Tokai University Kitakaname Hiratsuka-shi Kanagawa 259-1292 Japan; d School of Science, Kwansei Gakuin University 1 Gakuen Uegahara Sanda Hyogo 669-1330 Japan; e Frontier Research Institute for Interdisciplinary Sciences, Tohoku University 6-3 Aramaki-Aza-Aoba, Aoba-ku Sendai 980-8578 Japan okmasaki@tohoku.ac.jp; f Institute of Global Innovation Research, Tokyo University of Agriculture and Technology 3-8-1 Harumi-cho Fuchu Tokyo 183-8538 Japan; g Kanagawa Institute of Industrial Science and Technology 3-2-1 Sakato, Takatsu-ku Kawasaki Kanagawa 213-0012 Japan

## Abstract

We report the first example of a synthetic thiol-based compound that promotes oxidative protein folding upon 1-equivalent loading to the disulfide bonds in the client protein to afford the native form in over 70% yield. *N*-Methylation is a central post-translational processing of proteins *in vivo* for regulating functions including chaperone activities. Despite the universally observed biochemical reactions in nature, *N*-methylation has hardly been utilized in the design, functionalization, and switching of synthetic bioregulatory agents, particularly folding promotors. As a biomimetic approach, we developed pyridinylmethanethiols to investigate the effects of *N*-methylation on the promotion of oxidative protein folding. For a comprehensive study on the geometrical effects, constitutional isomers of pyridinylmethanethiols with *ortho*-, *meta*-, and *para*-substitutions have been synthesized. Among the constitutional isomers, *para*-substituted pyridinylmethanethiol showed the fastest disulfide-bond formation of the client proteins to afford the native forms most efficiently. *N*-Methylation drastically increased the acidity and enhanced the oxidizability of the thiol groups in the pyridinylmethanethiols to enhance the folding promotion efficiencies. Among the isomers, *para*-substituted *N*-methylated pyridinylmethanethiol accelerated the oxidative protein folding reactions with the highest efficiency, allowing for protein folding promotion by 1-equivalent loading as a semi-enzymatic activity. This study will offer a novel bioinspired molecular design of synthetic biofunctional agents that are semi-enzymatically effective for the promotion of oxidative protein folding including biopharmaceuticals such as insulin *in vitro* by minimum loading.

## Abstract 2

We report the first example of a synthetic thiol-based compound that promotes oxidative protein folding upon 1-equivalent loading to the disulfide bonds in the client protein to afford the native form in over 70% yield.

## Graphical abstract

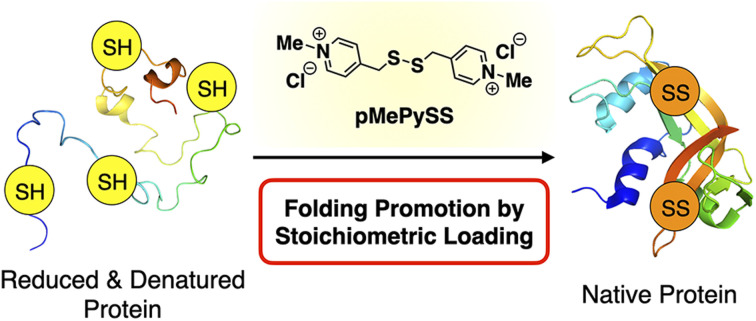

## Introduction

Methylation is one of the simplest and most commonly observed structural modifications of biomacromolecules to regulate their functions. In proteins, posttranslational methylation occurs at several amino acid residues such as lysine, arginine, and histidine.^1,2^ Methylation at a heteroatom in a side chain supplies the amino acid residue with a cationic charge to alter the electronic characteristics at the peripheral and even distantly positioned domains, upon which the protein changes its conformation, localization in the cell, and interactions with partner molecules and substrates. Chaperones, proteins that assist protein folding, are known to be regulated by methylation. Hsp90, for example, is a highly conserved molecular chaperone in the cytoplasm of eukaryotic cells, and its C-terminal domain can be methylated by a methyltransferase.^3–5^ The methylation leads to a conformational change and enhances interaction with a client protein. Such “methylation-regulated” functional control inspired us to design biomimetic synthetic folding promotors responsive to methylation.

**Fig. 1:**
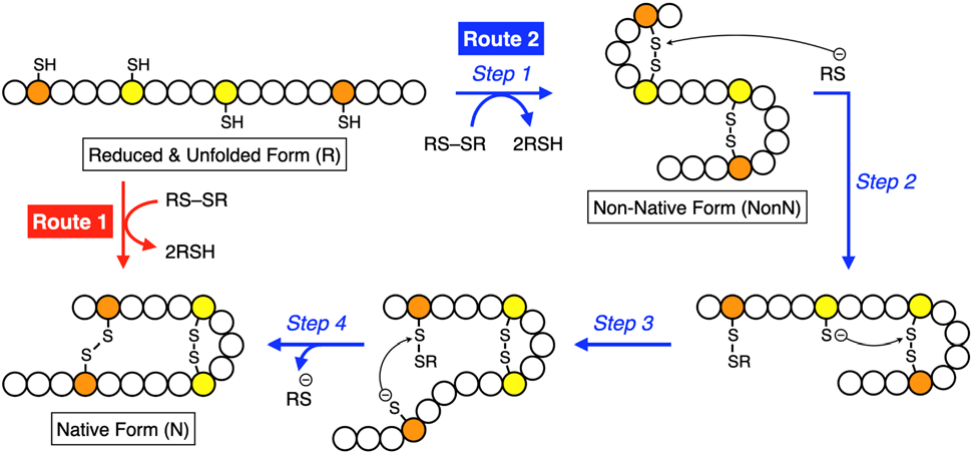
Schematic illustration of oxidative folding of a polypeptide chain forming two disulfide bonds. Orange and yellow circles represent cysteine residues that form disulfide bonds between the same color pairs, and white circles represent amino acid residues other than cysteine. Route 1: oxidation reaction directly from a reduced and unfolded form (R) to the native form (N). Route 2: oxidation reaction (step 1) followed by disulfide bond isomerization (steps 2–4) from R to N *via* non-native form (NonN).

Folding is an essential reaction of proteins to express their bioactivities by forming native conformations (N).^6,7^ Among the folding reactions, oxidative protein folding is a process that includes disulfide (SS) bond formation between cysteine residues in the polypeptide chain during the transition from the reduced and unfolded state (R) to N under the thermodynamic or kinetic control (Fig. 1).^8–14^ An R-form polypeptide chain possessing multiple cysteine residues can proceed through several pathways of folding. One is a route that can form N by the straightforward reaction with an oxidant (RS-SR, route 1 in Fig. 1), while the other routes include the formation of a non-native form (NonN) with SS bonds between non-native cysteine pairs (route 2). The subsequent SS bond isomerization process allows for the conversion of NonN to N while gaining increased thermodynamic stability by cleavage and re-formation of SS bonds by inter- and intramolecular nucleophilic attacks of thiolate anions (steps 2–4 in route 2). In cells, redox-active enzymes belonging to the protein disulfide isomerase (PDI) family assist the oxidative protein folding by facilitating the formation and isomerization of SS bonds in the client proteins.^15–18^ In PDI, N- and C-terminal thioredoxin- (Trx-) like domains contain redox-functional active centers with the sequence CXHC.^19^ One of the notable properties of the CXHC motif is the low p*K*_a_ of the thiol (SH) groups. The high acidity of the SH groups allows for efficient production of thiolate (S^−^) species reactive for SS-bond isomerization in physiological conditions. The imidazolyl group, an aromatic base, in the histidine residue of the CPHC motif of prokaryotic disulfide isomerase plays a critical role in increasing the acidity of SH groups in the neighboring Cys residues.^20,21^

Inspired by the active sites, synthetic mimics of disulfide isomerases have been developed based on redox-active small molecules. Thiols or selenols conjugated with basic units are an established molecular structure as effective folding promotors.^22–29^ For example, glutathione (GSH) is known to promote oxidative protein folding, and the replacement of its glutamate residue with arginine leads to a significant acceleration of the folding reaction.^30^ A conjugate between a diselenide and a basic amino acid residue such as histidine is effective for the assistance of protein folding.^31^ It has been also reported that thiols bearing an amino or a guanidyl group prompt oxidative folding.^32,33^ Importantly, in these previous examples, the synthetic folding-promoting thiols were added in excess amounts relative to the client proteins, likely due to insufficient disulfide bond isomerization and anti-aggregation activities. Although developing small folding promotors functioning by enzymatic or stoichiometric loading is advantageous in industrial applications of protein synthesis and biological applications for redox-based cellular controls, effective molecular design of small thiol compounds with sufficient activities for minimum loading has not been reported. In this study, inspired by the biological methylation-triggered controls of protein activities, we demonstrate the remarkable acceleration of oxidative protein folding facilitated by *N*-methylation of heteroaromatic thiols with semi-enzymatic efficiencies.

## Results and discussion

### Molecular design and chemical characterizations

**Fig. 2:**
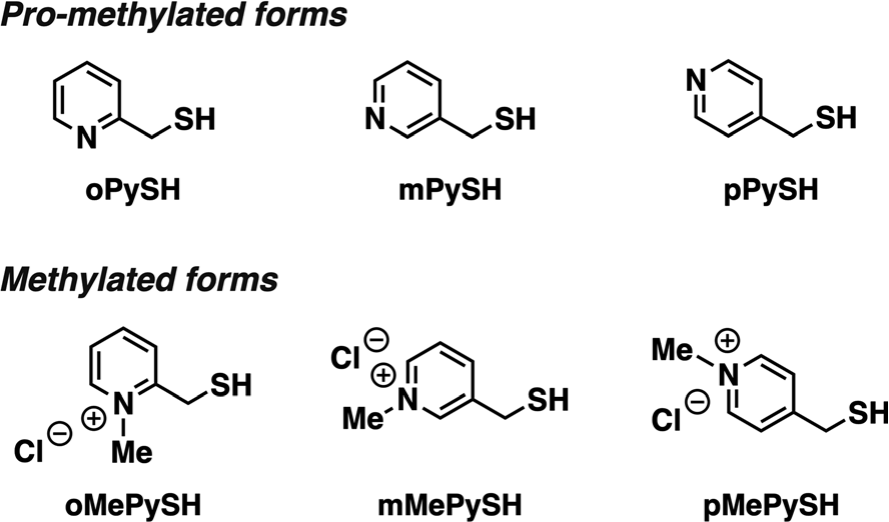
Molecular structures of oPySH, mPySH, and pPySH as pro-methylated forms and oMePySH, mMePySH, and pMePySH as methylated forms.

To investigate the effects of *N*-methylation of heteroaromatic thiols, we designed pyridinylmethanethiols (PySHs) as pro-methylated forms and their *N*-methylated forms (MePySHs, Fig. 2). *Ortho*-, *meta*- and *para*-substituted PySHs (oPySH, mPySH, pPySH) were designed to study the geometrical effects comprehensively. PySHs were synthesized from the corresponding (chloromethyl)pyridines. PySHs were oxidized with H_2_O_2_ to afford their disulfide forms, which were reacted with iodomethane for *N*-methylation. By subsequent reduction with dithiothreitol, MePySHs were successfully synthesized (oMePySH, mMePySH, pMePySH).

By monitoring the absorption intensity at 240 nm corresponding to thiolate anions at variable pH, p*K*_a_ values of the thiol groups of PySHs and their methylated forms were characterized (Table 1, Fig. S1 in ESI†). Interestingly, p*K*_a_ values of PySHs were commonly lower than that of GSH (9.17),^34^ likely due to their thiol-base conjugated structures, and pPySH showed the lowest p*K*_a_ value (oPySH: 9.04; mPySH: 9.05; pPySH: 8.68). Of importance, *N*-methylation further lowered the p*K*_a_ values of the thiol groups (oMePySH: 6.23; mMePySH: 7.64; pMePySH: 7.34). Redox potential *E*°′ is an indicator of the reducing power of a thiol group; higher *E*°′ indicates weaker reductant. In other words, the corresponding disulfide form possesses a higher oxidizing capability. PySHs and MePySHs, except oPySH, commonly showed higher *E*°′ values than GSH (Table 1, Fig. S2 in ESI†).^35^ The *E*°′ values of MePySHs were also higher than those of the corresponding PySHs.

**Table 1:** Chemical properties of PySHs and MePySHs

	p*K*_a_	*E*°′ (mV)		p*K*_a_	*E*°′ (mV)
oPySH	9.04 ± 0.08	−270 ± 1.5	oMePySH	6.23 ± 0.01	−204 ± 5.7
mPySH	9.05 ± 0.13	−250 ± 2.5	mMePySH	7.64 ± 0.03	−231 ± 1.3
pPySH	8.68 ± 0.05	−246 ± 0.4	pMePySH	7.34 ± 0.01	−211 ± 2.0
GSH	9.17 ± 0.12^a^	−256 ± 5^b^	—		

a Ref. 34.

b Ref. 35.

### Folding promotion of ribonuclease A by PySHs and MePySHs

**Fig. 3:**
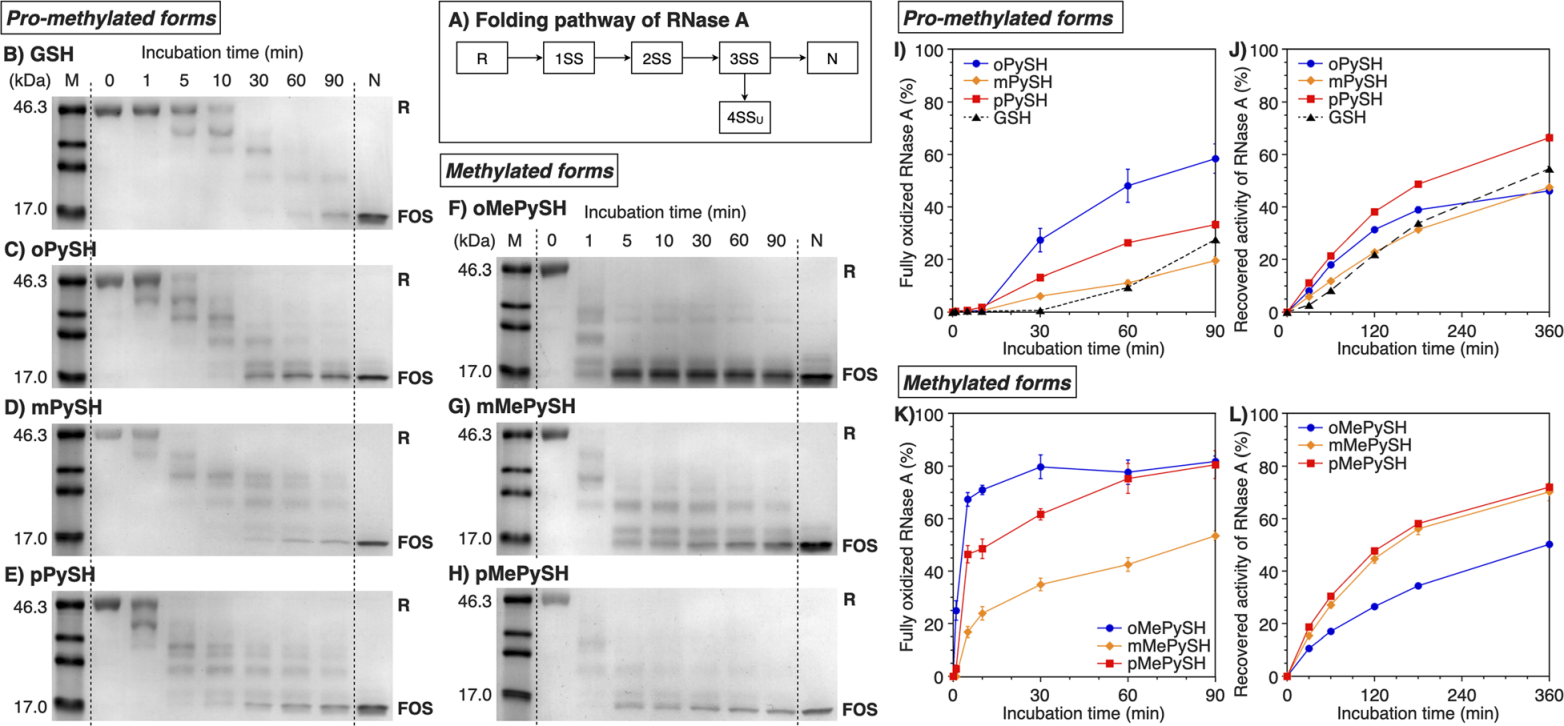
Time course analyses of RNase A oxidation by SDS-PAGE. (A) Folding pathway of RNase A. SDS-PAGE gel images monitoring the oxidation of RNase A (8.0 μM) in the presence of GSSG (0.20 mM) and (B) GSH, (C) oPySH, (D) mPySH, (E) pPySH, (F) oMePySH, (G) mMePySH and (H) pMePySH (thiol compounds: 1.0 mM) in a buffer (50 mM Tris–HCl, 300 mM NaCl, pH 7.5) at 30 °C. The folding reactions were quenched with malPEG-2000 after 1, 5, 10, 30, 60 and 90 min incubations. The leftmost and rightmost lanes indicate the bands corresponding to the markers (M) and native RNase A (N), respectively. R and FOS denote fully reduced and fully oxidized species of RNase A, respectively. Time-course changes of (I and K) formation of fully oxidized RNase A (4SS forms, 8.0 μM) and (J and L) recovered enzymatic activity of RNase A (8.0 μM) in the presence of GSSG (0.20 mM) and (I and J) oPySH, mPySH, pPySH and GSH (1.0 mM) and (K and L) oMePySH, mMePySH and pMePySH (1.0 mM) in a buffer (50 mM Tris–HCl, 300 mM NaCl, pH 7.5) at 30 °C quantified by SDS-PAGE analyses (original data in C–H). The activity was evaluated by spectroscopic monitoring of the hydrolysis of cCMP to 3′-CMP at 30 °C. Error bars indicate the means ± SEM of three independent experiments.

To study the folding promotion effects of the PySHs and their methylated forms, SS bond formation and enzymatic activity recovery of reduced and unfolded ribonuclease (RNase) A were monitored. RNase A is a representative protein used in oxidative folding assays, possessing four disulfide bonds between cysteine residues at C26–C84, C40–C95, C58–C110, and C65–C72.^10,36–38^ As schematically indicated in Fig. 3A, reduced and unfolded RNase A (R) forms SS bonds in a consecutive manner during the folding process. Importantly, in addition to N, RNase A can form non-native conformations (4SS_U_) as fully oxidized species (FOS) with four intramolecular disulfide bonds. To monitor the progress of disulfide-bond formation of RNase A, the folding reaction was quenched by adding malPEG-2000 (average *M*_n_ = 2000) at selected time points. The maleimide group of malPEG-2000 couples with thiol groups irreversibly to increase the mass of the protein, allowing for the separation of R, intermediates, and FOS of RNase A by sodium dodecyl sulfate-polyacrylamide gel electrophoresis (SDS-PAGE). Since disulfides of the pro-methylated pyridinylmethanethiols (PySSs) were poorly soluble in water, GSSG was used as an oxidant to propel the folding reaction. In the presence of oPySH and GSSG, the band intensities of R decreased over the incubation time, while the band corresponding to FOS emerged after 30 min incubation and further intensified as the incubation continued ([RNase A] = 8.0 μM; [GSSG] = 0.20 mM; [oPySH] = 1.0 mM; Fig. 3C). At 30 min incubation, RNase A showed a stronger band of FOS in the presence of oPySH than GSH in the SDS-PAGE analysis; therefore, it is indicated that the oPySH/GSSG system has a higher acceleration effect of SS bond formation than the GSH/GSSG system. Based on the electrophoretic assays (Fig. 3B–E), quantitative time-course profiles of RNase A FOS formation indicate that the oPySH/GSSG system showed the fastest SS-bond formation and the reaction rate decreased in the order of the pPySH/GSSG and mPySH/GSSG systems (Fig. 3I). Interestingly, in the recovery rate of the enzymatic activity of RNase A to monitor the formation of its native form, the pPySH/GSSG system showed the highest efficiency among the pro-methylated PySHs (Fig. 3J).

By *N*-methylation of PySHs, characteristics of these compounds were emphasized more sharply. The oMePySH/GSSG system converted 67% of RNase A to FOS within 10 min incubation, and the conversion was further enhanced to 80% by extending the incubation longer than 30 min (Fig. 3F and K). The FOS formation rate became slower in the order of oMePySH, pMePySH, and mMePySH in common with their pro-methylated forms (Fig. 3F–H and K). In the enzymatic activity monitoring, on the other hand, the addition of mMePySH and pMePySH showed significantly faster recovery rates than that of oMePySH (Fig. 3L). The rate constants for RNase A refolding were determined to be *k*_GSH_ = 2.7 × 10^−3^ min^−1^, *k*_oMePySH_ = 7.7 × 10^−3^ min^−1^, and *k*_pMePySH_ = 8.5 × 10^−3^ min^−1^ (calculated by IGOR Pro), *i.e.*, the reaction rate using pMePySH was more than three times faster than that using the typical glutathione system. Given that the enzymatic activity exclusively originated from the native form, the RNase A refolding in the presence of oMePySH should be highly error-prone, possibly producing oxidized non-native forms. Thus, *N*-methylation of pyridinylmethanethiols commonly accelerated the FOS formation of RNase A compared to the corresponding pro-methylated forms, and mMePySH and pMePySH demonstrated efficient refolding promotion of RNase A to the native form.

### Folding promotion of bovine pancreatic trypsin inhibitor by PySHs and MePySHs

**Fig. 4:**
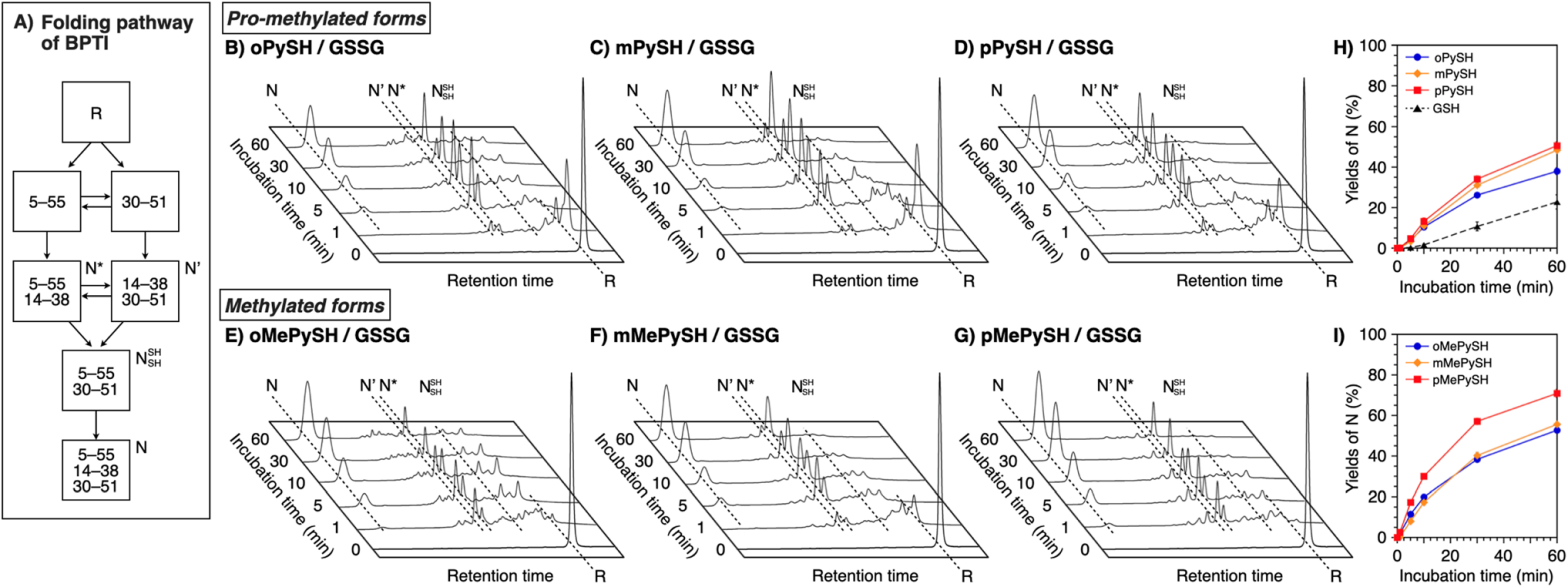
(A) Folding pathway of BPTI. Time-course reverse-phase HPLC analyses of oxidative folding of BPTI (30 μM) in the presence of GSSG (0.20 mM) and (B) oPySH, (C) mPySH, (D) pPySH, (E) oMePySH, (F) mMePySH and (G) pMePySH (1.0 mM) between 16 and 41 min retention time. N and R depict native and reduced forms of BPTI, respectively. Eluent buffers: water (containing 0.05% TFA) and CH_3_CN (containing 0.05% TFA) with a linear gradient; flow rate: 1.0 mL min^−1^; detection wavelength: 229 nm; temperature: 30 °C. Time course plots of the yields of N-BPTI in the presence of (H) oPySH, mPySH, pPySH, and GSH (based on (B–D)) and (I) oMePySH, mMePySH, and pMePySH (based on (E–G)). Error bars indicate the means ± SEM of three independent experiments.

To verify the generality of the folding acceleration by the *N*-methylation of pyridinylmethanethiols, the folding reaction of bovine pancreatic trypsin inhibitor (BPTI) was investigated in the presence of GSSG as an oxidant and the synthesized thiol compounds ([BPTI] = 30 μM; [GSSG] = 0.20 mM; [thiol] = 1.0 mM). N-BPTI contains three disulfide bonds at C5–C55, C14–C38, and C30–C51. The folding process of BPTI from its R-form proceeds *via* quasi-native intermediates, *i.e.*, N′, N*, and N^SH^_SH_ (Fig. 4A),^39^ that can be monitored quantitatively by reversed-phase high performance liquid chromatography (HPLC), useful for the time-course analysis of the folding progress. The HPLC analysis showed that the fraction of R-BPTI disappeared within 10 min of the incubation in the presence of oPySH/GSSG, and in the meantime, the fractions corresponding to the intermediates and N-BPTI appeared and intensified to reach 38% yield of N-BPTI after 60 min incubation (Fig. 4B and H). Interestingly, mPySH/GSSG and pPySH/GSSG systems afforded increased yields of N-BPTI after 60 min incubation (mPySH/GSSG: 48%; pPySH/GSSG: 51%; Fig. 4C, D and H). Importantly, the *N*-methylated thiols prompted the folding of BPTI more efficiently. In the oMePySH/GSSG system, the fraction of R-BPTI disappeared within 1 min incubation, and notably, formation of N-BPTI was visualized after the initial 1 min incubation. The area of the N-BPTI fraction increased upon extension of the incubation time to provide 53% yield after 60 min incubation (Fig. 4E and I). Further enhanced BPTI folding promotion was demonstrated by the pMePySH/GSSG system. R-BPTI was rapidly oxidized within 1 min incubation, at which the fraction of N-BPTI appeared. By extending the incubation up to 60 min, the yield of N-BPTI increased to 71% (Fig. 4G and I).

### Discussion of chemical properties of PySHs and MePySHs on the folding process

As demonstrated in the RNase A and BPTI folding assays, the *N*-methylation significantly enhanced the yields of the native-structure formation. HPLC analysis indicated that, in the preincubated mixture of pMePySH and GSSG, pMePySS and pMePyS-SG were generated (Fig. S3†). It is likely that pMePySS and pMePyS-SG generated *in situ* react with a protein as oxidants to facilitate the disulfide-bond formation, therefore the oxidation of the proteins is accelerated (step 1 in Fig. 1). The added thiol compound, pMePySH, accelerates the disulfide-bond isomerization by an intermolecular nucleophilic attack to form pMePyS–Cys and Cys exposing a free thiol group, and the regenerated Cys can attack another Cys–Cys by an intramolecular nucleophilic reaction (steps 2–4 in Fig. 1). The disulfide-bond isomerization should allow for conformational changes of the protein to gain increased thermodynamic stability to lead to the folding into its native structure. Therefore, higher oxidizability and nucleophilicity of pMePySS and pMePySH, respectively, accelerate the overall oxidization of the client proteins to afford FOS and enhances the shuffling efficiency to promote the folding to the native form.

In the RNase A folding assays, disulfide-bond formation to afford FOS proceeded faster in the presence of MePySHs/GSSG than PySHs/GSSG (Fig. 3I and K), where MePySS and PySS would be presumably generated in the preincubated mixture for acting as oxidants. The rapid disappearance of the R-BPTI fractions within 1 min incubation also demonstrates the faster disulfide-bond formation in the presence of MePySHs/GSSG (Fig. 4E–G). The faster disulfide-bond formation by MePySHs than PySHs would be attributed to their higher *E*°′. The lower p*K*_a_ of MePySHs than the corresponding PySHs should be advantageous for the nucleophilic attack to the Cys–Cys bonds to facilitate disulfide-bond isomerization. Here, it may look contradictory that oPySH showed the fastest disulfide-bond formation of RNase A despite its significantly low *E*°′ value. The fastest disulfide bond formation by oPySH could be attributed to the formation of intermolecular disulfide bonds between oPySH and cysteine residues of RNase A, which would be stabilized by the reductive property of oPySH.^40^

Among MePySHs, it is characteristic that oMePySH propelled the rapid disulfide bond formation of RNase A and BPTI. Irrespective of the rapid disulfide bond formation, oMePySH showed the lowest efficiency of refolding to the native forms among MePySHs (Fig. 3L and 4I). One critical reason for the low folding promotion efficiency is the formation of non-native and off-pathway intermediates in the presence of oMePySH. Formation of the non-native and off-pathway species is indicated in Fig. 4E, where BPTI showed numerous fractions between the fractions of N* and R after 1 min incubation and a complex fraction pattern was observed between the N- and N′-fractions even after 60 min incubation. The long retention of the non-native and off-pathway species of BPTI over the 60 min incubation process suggests low SS-bond shuffling capability of oMePySH. The nucleophilicity of the thiol group of oMePySH would be reduced possibly by the steric hindrance with the methyl group close to the thiol group and stabilization of the thiolate anion by the neighboring cationic nitrogen atom. As a result, pMePySH showed superior performance in the promotion of oxidative protein folding to prompt disulfide bond formation and afford the native forms in the highest yields. It is likely that the higher *E*°′ and lower p*K*_a_ values of pMePySH than mMePySH allowed for the faster disulfide bond formation and efficient disulfide bond shuffling to provide the native form proteins effectively (Fig. 3L, 4F and G).

### Semi-enzymatic folding promotion by pMePySH/pMePySS

In the endoplasmic reticulum where oxidative folding mainly occurs, PDI plays a central role in the assistance of oxidative protein folding.^15–18^ Due to the molecular recognition property, PDI monomer or its dimer binds with a substrate protein to prompt the formation and shuffling of disulfide bonds directed to forming the native structures along the client folding pathway.^41^ In contrast to such “one-to-one” enzymatic reactions between PDI and substrates, conventional low-molecular-weight compounds such as glutathione are commonly used on the mM-order against several μM substrates for protein folding promotion.^41,42^ The high performance of pMePySH inspired us to attempt an oxidative protein folding promotion under a more biomimetic condition, *i.e.*, protein folding in the presence of only 1-equivalent of an additive to the number of disulfide bonds in a client protein. Surprisingly, even in such a semi-enzymatic condition, highly efficient protein folding promotion could be demonstrated by the *N*-methylated compound.

**Fig. 5:**
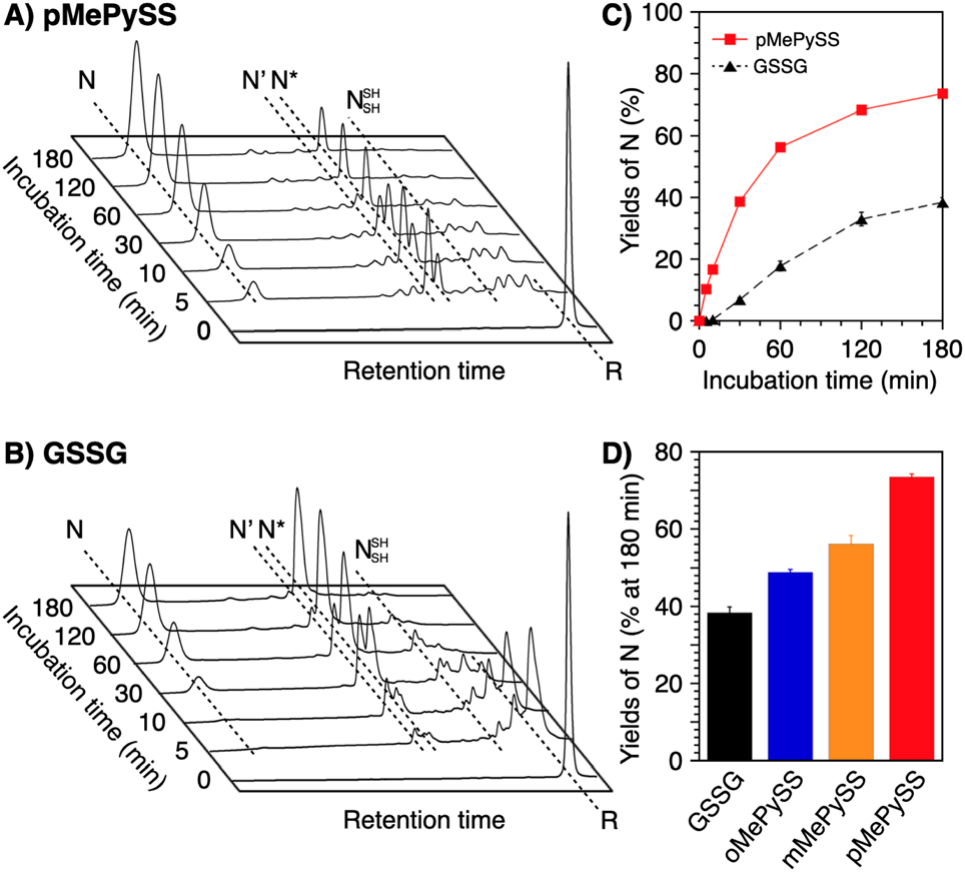
Time-course reverse-phase HPLC analyses of oxidative folding of BPTI (30 μM) in the presence of (A) pMePySS (90 μM) and (B) GSSG (90 μM) between 16 and 41 min retention time. N and R depict native and reduced forms of BPTI, respectively. Eluent buffers: water (containing 0.05% TFA) and CH_3_CN (containing 0.05% TFA) with a linear gradient; flow rate: 1.0 mL min^−1^; detection wavelength: 229 nm; temperature: 30 °C. (C) Time course plots of the yields of N-BPTI in the presence of pMePySS and GSSG (based on (A) and (B)). (D) A bar graph indicating the yields of N-BPTI after 180 min incubation in the presence of GSSG, oMePySS, mMePySS, and pMePySS. Error bars indicate the means ± SEM of three independent experiments.

For this semi-enzymatic assay, we chose pMePySS, the disulfide form of pMePySH, because of its sufficient water-solubility, high oxidizability, and the productivity of pMePySH, which showed the highest folding promotion performance by encouraging SS-bond shuffling after the oxidation reaction. We added 90 μM pMePySS to R-BPTI (30 μM) as the minimum loading for oxidizing the six cysteine residues in BPTI to form three disulfide bonds. HPLC analysis indicated the disappearance of R-BPTI and formation of N-BPTI within the initial 5 min incubation, suggesting rapid progress of the oxidation reaction. After 60 min incubation, N-BPTI formed in 56% yield, and the yield was further enhanced to 74% after 180 min incubation (Fig. 5A and C). In contrast, the oxidation of R-BPTI proceeded significantly slower over 30 min, and formation of N-BPTI was much less efficient in the presence of oxidized glutathione (GSSG, 90 μM) compared to pMePySS. The HPLC analysis indicated that the yield of N-BPTI was limited to only 38% even after 180 min incubation (Fig. 5B). As expected, among the constitutional isomers of MePySSs, pMePySS showed the most efficient protein folding promotion in the 1-equivalent-loading semi-enzymatic condition (Fig. 5D and S4 in ESI†). The *N*-methylated pyridinylmethanethiols also accelerated the folding of RNase A in the semi-enzymatic condition (Fig. S5 in ESI†). Furthermore, to evaluate the practical significance, we investigated the folding promotion of human insulin, a therapeutic protein, by pMePySS. Insulin comprises two peptide chains (A- and B-chains) with two interchain disulfide bridges and one intrachain disulfide bond in A-chain; therefore, its folding velocity and efficiency are essentially low.^43^ Importantly, under a catalytic condition, pMePySS (200 μM) showed increased efficiency of the chain-coupling of A- and B-chains (200 μM each) of human insulin to afford its native form with a 4.2-fold higher yield relative to GSSG (Fig. S6 and S7 in ESI†). Hence, high activation of thiols for the protein folding promotion was demonstrated by the *N*-methylation of the pyridinyl group; and particularly pMePySH and its oxidized form promoted and accelerated the oxidative protein folding efficiently even by stoichiometric amount loading.

## Conclusions

We have demonstrated the remarkable *N*-methylation effects of heteroaromatic thiols on the promotion of oxidative protein folding. *N*-Methylation is a ubiquitous and central post-translational processing of proteins for their functional regulation including chaperone activities. Although it has been revealed that methylation at a histidine residue is involved in the switching of chaperone functions such as Hsp90, there are few examples of research on the regulation of chaperone function by methylation, and much remains elusive. Despite the universally observed biochemical reactions in nature, *N*-methylation has hardly been utilized in the design, functionalization and switching of synthetic bioregulatory agents, particularly in the development of folding promotors. Based on this comprehensive study of PySHs including all constitutional isomers and their *N*-methylated forms revealing the geometrical dependency on the chemical properties, we demonstrated highly efficient folding promotion by pMePySH. Chemical characterizations indicated that *N*-methylation enhances both acidity and oxidizability of the thiol groups, which likely enabled the remarkable enhancement of the folding reactions by accelerated SS-bond formation and effective shuffling. Using pMePySS, we successfully demonstrated the first example of the semi-enzymatic promotion of oxidative protein folding upon 1-equivalent loading of the chemical oxidant, which afforded the native form protein in over 70% yield. The development of small chemical compounds that assist and promote protein folding is critically important not only in basic biological studies for the synthesis of natural and mutant proteins but also in industrial applications to produce protein pharmaceuticals such as insulin. Of note, enzymatic dysfunction such as PDI can result in pathological outcomes such as neurodegenerative disease and diabetes.^44^ The chemical structure-based design for semi-enzymatic agents that can replace the enzymes may thus give clues to new approaches for preventing enzyme-related pathogenic protein aggregation. This study will offer a chemical viewpoint of methylation effects on the regulation of enzymatic activities as well as an effective bioinspired molecular design of functional compounds that are useful for the promotion of oxidative protein folding *in vitro* by minimum loading.

## Data availability

Information supporting this article has been uploaded as part of the ESI.† Additional data are available from the authors on reasonable request.

## Author contributions

T. M. conceived the idea of this study. S. O., Y. M., R. T., K. A., S. K., and M. O. performed the experiments. M. O. and T. M. supervised the conduct of this study. S. O., M. O., and T. M. wrote the manuscript. All authors critically reviewed and revised the manuscript draft and approved the final version for submission.

## Conflicts of interest

There are no conflicts to declare.

## Supplementary Material

SC-014-D3SC01540H-s001
