# Supplementary material for: Semi-enzymatic acceleration of oxidative protein folding by N-methylated heteroaromatic thiols
Source: Chem Sci. 2023 Jun 16;14(28):7630–6. doi: 10.1039/d3sc01540h (PMC10355094; doi:10.1039/d3sc01540h)
Supplement: SC-014-D3SC01540H-s001 [file SC-014-D3SC01540H-s001.pdf]

Electronic Supplementary Material (ESI) for Chemical Science.
This journal is © The Royal Society of Chemistry 2023

Supplementary Information

Semi-enzymatic acceleration of oxidative protein folding by *N*-methylation of heteroaromatic thiols

Shunsuke Okada,[a†] Yosuke Matsumoto,[a†] Rikana Takahashi,[b] Kenta Arai,[b,c]
Shingo Kanemura,[d] Masaki Okumura*[e] and Takahiro Muraoka*[a,f,g]

[a] *Department of Applied Chemistry, Graduate School of Engineering, Tokyo University of Agriculture and Technology, 2-24-16 Naka-cho, Koganei, Tokyo 184-8588, Japan.*
E-mail: muraoka@go.tuat.ac.jp

[b] *Department of Chemistry, School of Science, Tokai University, Kitakaname, Hiratsuka-shi, Kanagawa, 259-1292 Japan.*

[c] *Institute of Advanced Biosciences, Tokai University, Kitakaname, Hiratsuka-shi, Kanagawa, 259-1292 Japan.*

[d] *School of Science, Kwansei Gakuin University, 1 Gakuen Uegahara, Sanda, Hyogo 669-1330, Japan.*

[e] *Frontier Research Institute for Interdisciplinary Sciences, Tohoku University, 6-3 Aramaki-Aza-Aoba, Aoba-ku, Sendai 980-8578, Japan.*
E-mail: okmasaki@tohoku.ac.jp

[f] *Institute of Global Innovation Research, Tokyo University of Agriculture and Technology, 3-8-1 Harumi-cho, Fuchu, Tokyo 183-8538, Japan.*

[g] *Kanagawa Institute of Industrial Science and Technology, 3-2-1 Sakato, Takatsu-ku, Kawasaki, Kanagawa 213-0012, Japan.*

† These authors contributed equally to this study.

Table of Contents

1. Materials S2
2. Instrumentation S3
3. Methods S4
4. Synthesis S9
5. HPLC analysis of a mixture of pMePySH and GSSG S16
6. HPLC analysis of BPTI folding S17
7. Enzymatic activity recovery of RNase A S18
8. HPLC analysis of insulin folding S19
9. ELISA analysis of insulin S21
10. Supplementary references S22

1. Materials

Deuterated solvents were purchased from Kanto Chemicals (Tokyo, Japan). Acetonitrile, *tert*-butyl methyl ether, 30% H_2O_2 aq., 35% hydrochloric acid, 2-propanol, sodium chloride, sodium hydroxide (NaOH), thiourea and trifluoroacetic acid (TFA) were purchased from Kishida Chemical (Tokyo, Japan). Coomassie brilliant blue G-250, 5,5'-dithiobis(2-nitrobenzoic acid) (DTNB), 1,4-dithiothreitol (DTT), L-glutathione oxidized (GSSG), L-glutathione reduced (GSH) and guanidine hydrochloride (GdnHCl) were purchased from Nacalai Tesque (Kyoto, Japan). Bovine pancreatic trypsin inhibitor (BPTI) was purchased from Pro-Spec-Tany TechnoGene (Rehovot, Israel). Cytidine 2':3'-cyclic monophosphate monosodium salt (cCMP), maleimidePEG-2000 (malPEG-2000), and ribonuclease A (RNase A) from bovine pancreas were purchased from Sigma-Aldrich (St. Louis, MO, USA). 2-(Chloromethyl)pyridine hydrochloride, 3-(chloromethyl)pyridine hydrochloride, 4-(chloromethyl)pyridine hydrochloride and thiourea were purchased from Tokyo Chemical Industry (Tokyo, Japan). Dry acetonitrile and iodomethane were purchased from Kanto Chemicals. 2-Aminoethyl methanethiosulfonate (AEMTS) was synthesized by following the literature.[1] Human insulin A- and B-chains were prepared according to the previous method.[2] Column chromatography was carried out with Silica Gel 60 (spherical, neutral, particle size: 63–210 μm) purchased from Kanto Chemicals. Deionized water (filtered through a 0.22 μm membrane filter, >18.2 MΩ cm) was purified in Purelab DV35 of ELGA (Buckinghamshire, UK) and a Milli-Q system of Merck Millipore (Burlington, MA, USA).

2. Instrumentation

Nuclear magnetic resonance (NMR) spectra were recorded on a JNM-ECX 400 spectrometer (400 MHz for ^{1}H, 100 MHz for ^{13}C) of JEOL (Tokyo, Japan), where the chemical shifts (δ) were determined with respect to the resonances corresponding to the residual non-deuterated solvent for ^{1}H ($CDCl_3$: 7.24 ppm, D_2O: 4.67 ppm, CD_3OD: 3.29 ppm) and the resonances of the solvent for ^{13}C ($CDCl_3$: 77.16 ppm, CD_3OD: 47.68 ppm). High-resolution electrospray ionization (HR ESI) TOF MS spectra were recorded on micrOTOF-Q II-S1 of Bruker. Analytical thin layer chromatography (TLC) was performed on precoated, glass-backed silica gel Merck 60 F254. Visualization of the developed chromatogram was performed by UV absorbance, Hanessian's stain or iodine. UV-vis absorption spectra were recorded on V-750 UV-Vis spectrophotometer of JASCO (Tokyo, Japan). Analytical reversed-phase high-performance liquid chromatography (RP-HPLC) was conducted with Primaide HPLC system of HITACHI (Tokyo, Japan) using TSKgel Protein C4-300 column of Tosoh Bioscience (φ4.6 × 150 mm, Tokyo, Japan) for BPTI folding assay, and JASCO HPLC system using YMC Triart C18 column (φ4.6 × 250 mm, Kyoto, Japan) for redox potential measurements. Semi-preparative reversed-phase high-performance liquid chromatography performed on PU-4086-Binary pump, UV-4075 detector and CHF122SC fraction collector of JASCO (Tokyo, Japan) attached with TA12S05-2520WX Actus Triart column of YMC (φ20 × 250 mm, Tokyo, Japan).

3. Methods

1) Determination of thiol concentration for oxidative protein folding assay:

A thiol compound dissolved in 10 mM HCl aq. was diluted in a buffer (50 mM Tris-HCl, 0.3 M NaCl, pH 7.5). The mixture was added to an aqueous solution of 5,5'-dithiobis(2-nitrobenzoic acid) (DTNB). The concentration of the thiol compound was determined by the absorbance at 412 nm measured at 30 ºC with V-750 UV-Vis spectrophotometer.[3]

2) Preparation of reduced and denatured RNase A: RNase A was dissolved in a buffer (200 mM Tris-HCl, pH 8.7) containing 6.0 M GdnHCl and 100 mM DTT and the mixture was incubated for 2 h at 25 °C. The resulting mixture was dialyzed three times for 2 h each with 10 mM HCl aq. to remove the denaturing and reducing reagents. After the dialysis, the sample was stored at –30 ºC until use.

3) RNase A refolding assay: Prior to the assay, a mixture of GSSG and a thiol compound was dissolved in a buffer (50 mM Tris-HCl, 300 mM NaCl, pH 7.5), and the resulting mixture was preincubated for 10 min at 30 °C. To the preincubated mixture, fully reduced and denatured RNase A (8.0 μM) was added and incubated at 30 °C in a buffer (50 mM Tris-HCl, 300 mM NaCl, pH 7.5) containing 200 μM GSSG with 1.0 mM thiol compound.[4] At 30, 60, 120, 180 and 360 min after starting the incubation, aliquots (110 μL each) were taken from the reaction solution and were immediately added to a buffer (330 μL, 50 mM Tris-HCl, 300 mM NaCl, pH 7.5) containing cCMP (final concentration of cCMP = 0.60 mM) followed by the measurement of the linear increase in absorbance at 284 nm at 30 °C with V-750 UV-Vis spectrophotometer. Values represent means ± SEM based on the three independent experiments.

4) Gel shift assay of RNase A folding: Oxidative folding of RNase A (8.0 μM) was carried out in a buffer (50 mM Tris-HCl, 300 mM NaCl, pH 7.5) containing 200 μM GSSG with 1.0 mM thiol compound. At 0, 1, 5, 10, 30, 60 and 90 min after starting the incubation, free thiols were inactivated by the addition of Laemmli's 4×SDS-loading buffer[5] containing 10 mM malPEG-2000. RNase A was separated depending on the number of disulfide bonds by non-reducing 14% SDS-PAGE using WIDE RANGE gel (Nacalai Tesque, Kyoto, Japan). Proteins were visualized by coomassie brilliant blue staining. The gel image was imported with a

ChemiDoc Touch Imaging System and the band intensities were analyzed by Image Lab software (Bio-Rad, Hercules, CA, USA).

5) BPTI folding assay: BPTI (10 mg) dissolved in 0.1 M Tris (pH 8.0, 1 mL) containing 30 mM DTT and 8 M urea was incubated for 3 h at 50 °C. Fully reduced and denatured BPTI was purified by RP-HPLC using an InertSustain C18 column (φ4.6 × 250 mm, GL Sciences, Tokyo Japan), and the collected fractions were lyophilized. The obtained powder was stored at –30 °C until use. For the assay, a mixture of GSSG and a thiol compound was dissolved in a buffer (50 mM Tris-HCl, 300 mM NaCl, pH 7.5), and the resulting mixture was preincubated for 10 min at 30 °C. To the preincubated mixture, fully reduced and denatured BPTI (600 μM) dissolved in 6 M urea containing 0.05% trifluoroacetic acid was added (final concentration of BPTI: 30 μM, urea: 300 mM, GSSG: 200 μM, thiol compound: 1.0 mM, Tris-HCl: 50 mM, NaCl: 300 mM, pH 7.5). After the incubation at 30 °C for predetermined periods, the reaction was quenched by adding an equal volume of 1 M HCl aq., which was then analyzed by RP-HPLC at a flow rate of 1.0 mL min^{-1} monitoring at 229 nm with a linear gradient of elutions (solvent A: 0.05% trifluoroacetic acid in water; solvent B: 0.05% trifluoroacetic acid in acetonitrile; percentages of solvent A: 95% at 0 min, 80% at 15 min, 30% at 115 min).

6) Determination of thiol pK_a values: Stock solutions of citric buffer (sodium citrate and HCl for pH 2.0–4.0), phosphate buffer (Na_2HPO_4 and KH_2PO_4 for pH 5.0–8.0), borate buffers ($Na_2B_4O_7$ and HCl for pH 8.5–9.0, $Na_2B_4O_7$ and NaOH for pH 10.0–13.0) were degassed with N_2 for 1 h immediately prior to use. A stock solution of a thiol compound in degassed water (5.0 mM) were then prepared. Immediately after the aqueous solution of the thiol compound (50 μL) and a buffer (1.95 mL) were combined in a 1-cm thick quartz cuvette, the UV absorption spectrum of the sample was measured. The pH value of the sample was measured by a HORIBA pH meter (9618S-10D), which had been calibrated prior to use with pH 4.01, 6.86 and 9.18 standard solutions (HORIBA 101-S). Absorbance values at 240 nm of the samples were plotted in the function of the pH values, and the pK_a value of the thiol compound was calculated with KaleidaGraph software (version 5.0.3) by a curve fitting analysis using the following equation:

$$y = a + (\mathrm{m1})/(\mathrm{m2}*10\wedge(-x) + 1);\ \mathrm{m1} = b;\ \mathrm{m2} = 1000$$

where a is the absorbance of a sample below pH 3 and b is the difference of absorbances above the pH 11 and below the pH 3, and curve fitting calculation provides m2 = pK_a. For all analyses, r^2 values were higher than 0.99.

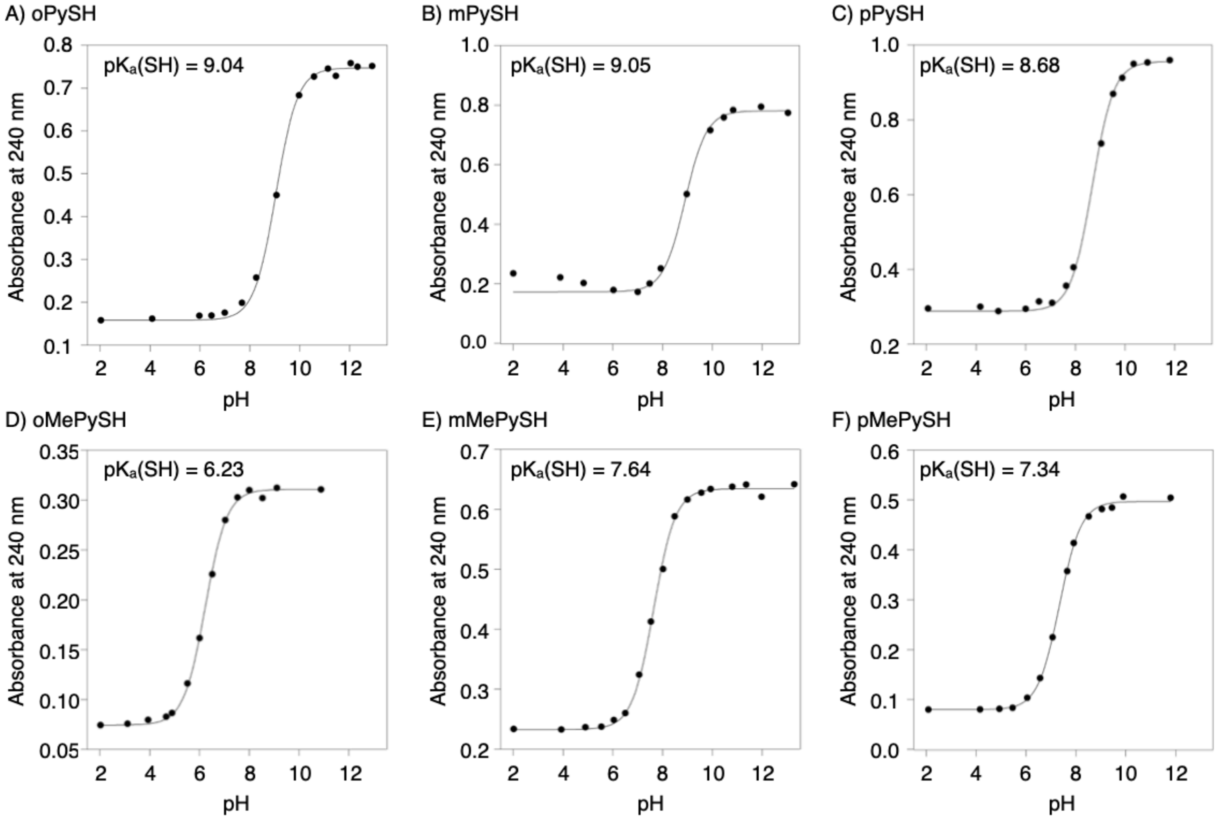

Fig. S1 Absorbance changes at 240 nm of (A) oPySH, (B) mPySH, (C) pPySH, (D) oMePySH, (E) mMePySH and (F) pMePySH at variable pH at 25 °C and curve fitting analyses.

7) Redox potential *E*°' measurements: *E*°' values of the thiol compounds were determined by following the protocol described in a previous paper.[6] A buffer (100 mM Tris-HCl, 1.0 mM EDTA, pH 7.0) was degassed with N_2 for longer than 1 h prior to use. DTT^{red} (60 µM, 4.9 mL) in the buffer was added to a disulfide (60 µM, 4.9 mL) in the buffer containing dimethyl sulfoxide (DMSO, final conc. = 0.5 v/v%) under N_2 at 25 ± 0.1 °C for 3 or 24 h (3 h for pyridinium-type disulfides, whereas 24 h for pyridine-type disulfides). Small amounts of DMSO were added to dissolve a pyridine-type disulfide. To quench the reaction, an aliquot of the reaction mixture (1 mL) was added to 1 M HCl aq. (200 µL), and the obtained solution was immediately analyzed by RP-HPLC (YMC Triart C18 column, φ4.6 × 250 mm). The RP-HPLC analysis was conducted with water/acetonitrile = 98/2 containing 0.1% TFA. The

concentrations of the species at equilibrium were calculated from the observed peak areas and the corresponding calibration curves.

The equilibrium constant K_{eq} for the reaction (eq. 1), described as eq. 2, was determined by averaging seven times of individual experiments following the above procedure.

$$\text{Disulfide} + \text{DTT}^{\text{red}} \rightleftharpoons 2\text{Thiol} + \text{DTT}^{\text{ox}} \qquad (1)$$

$$K_{eq} = \frac{[\text{Thiol}]^2[\text{DTT}^{\text{ox}}]}{[\text{Disulfide}][\text{DTT}^{\text{red}}]} \qquad (2)$$

The redox potential $E°'$ was calculated by the Nernst's equation (eq. 3)

$$E°' = E°'_{\text{DTT}} + \frac{RT}{nF} \ln K_{eq} \qquad (3)$$

where, n is the number of transferred electrons (n = 2), F is Faraday's constant (96500 C mol^{-1}), R is the universal gas constant (8.314 J K^{-1} mol^{-1}), T is the temperature (298 K), and $E^{0\prime}_{\text{DTT}}$ is the redox potential of DTT (–327 mV).

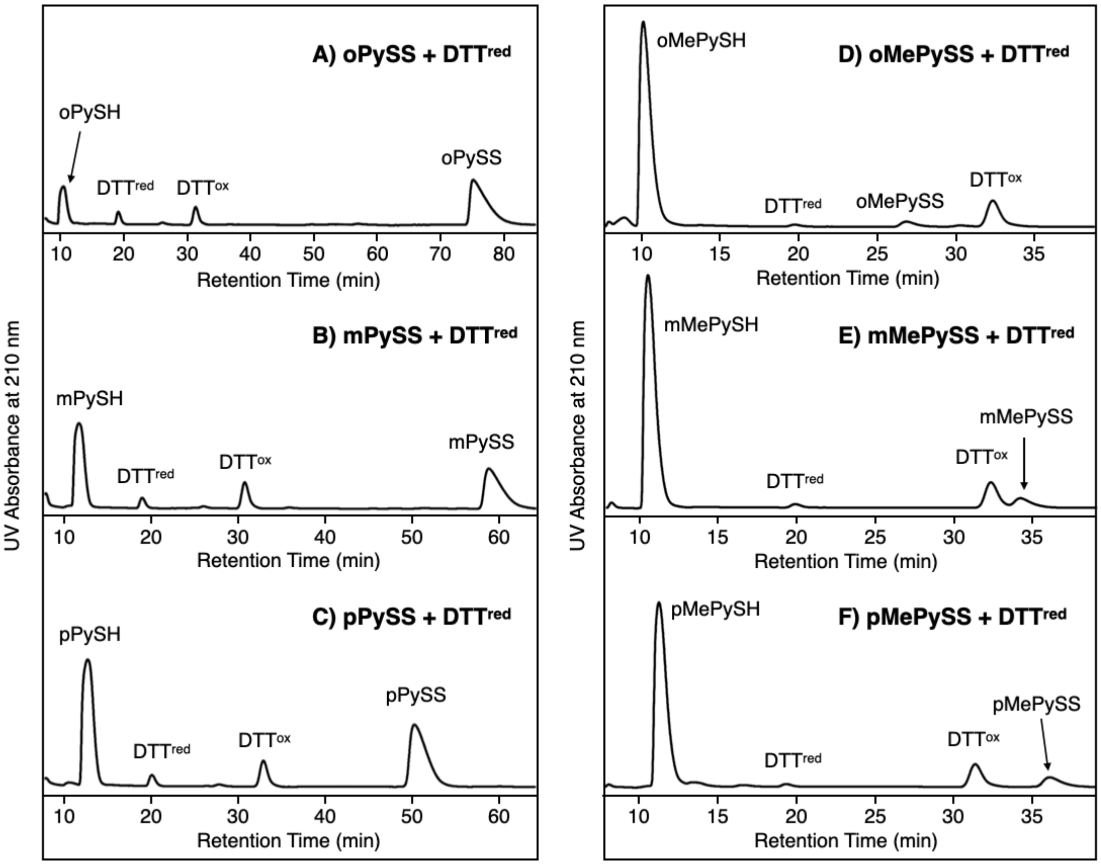

Fig. S2 Representative RP-HPLC traces of equilibrated mixtures of (A) oPySS, (B) mPySS, (C) pPySS, (D) oMePySS, (E) mMePySS, and (F) pMePySS with DTT^{red}. Reaction conditions were $[disulfide]^0 = [DTT^{red}]^0 = 30$ μM at 25 °C and pH 7.0 for 3 and 24 h.

8) Insulin two-chain folding assay: Two-chain folding of human insulin was conducted by following the previous method with slight modifications.[2] Briefly, A- and B-chains (50 nmol each) and pMePySS (50 nmol) were dissolved in a mixture of a buffer solution (25 mM sodium bicarbonate, 0.53 M urea, and 1.0 mM EDTA, pH 10.0) and ethylene glycol (9:1 [v:v]). The resulting sample solution was incubated at –10 °C. After a specific time point, an aliquot (5.0 μL) was added to an aqueous solution of AEMTS (7 mg mL^{-1}, 200 μL) as the thiol-blocking reagent. After 5 min, the sample solution was diluted with an aqueous TFA solution (0.1%, 830 μL), and then analyzed by reverse-phase HPLC. Analytical conditions were the same as described previously.[2]

4. Synthesis

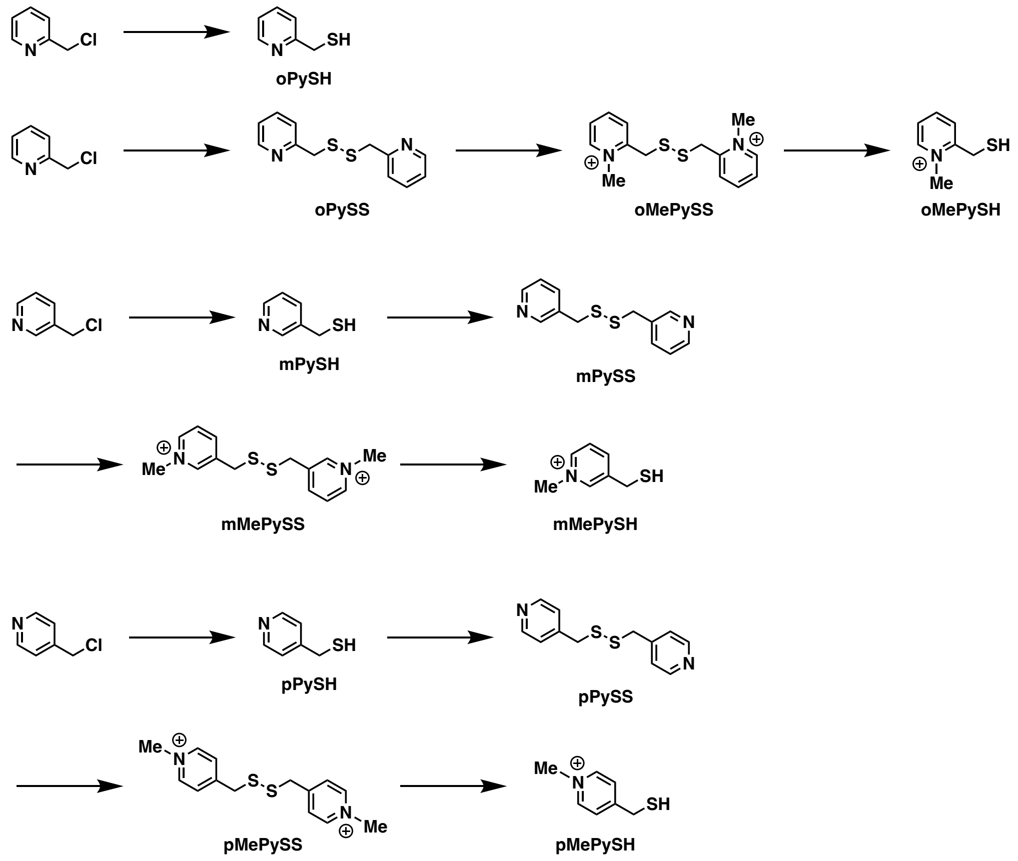

1) Synthesis of oPySH: To a degassed water solution (10 mL) of 2-(chloromethyl)pyridine (1.146 g, 6.987 mmol) was added thiourea (0.581 g, 7.64 mmol) under N_2, and the mixture was heated to 90 °C. After stirring for 2.5 h, to the mixture was added a degassed water (2 mL) solution of NaOH (0.840 g, 21.0 mmol). After stirring for additional 2.5 h at 90 °C, the reaction mixture was cooled to 25 °C. The mixture was washed out with *tert*-butyl methyl ether (10 mL, three times). The resulting aqueous mixture was neutralized to pH 7 with 1 M HCl aq. followed by extraction with CH_2Cl_2 (10 mL, three times). The organic extract was washed with brine (10 mL, once), dried over Na_2SO_4 and filtered off from insoluble substances through a filter paper. The filtrate was evaporated to dryness under reduced pressure, and the residue was chromatographed on silica gel with CH_2Cl_2 as an eluent to allow isolation of oPySH (0.508 g, 4.06 mmol) as colorless oil in 58% yield.

TLC (CH_2Cl_2) $R_f = 0.15$; ^{1}H NMR (400 MHz, $CDCl_3$, 25 °C): $\delta = 8.54$–8.52 (m, 1H), 7.64 (td, $J = 7.7$, 1.8 Hz, 1H), 7.32 (d, $J = 7.7$ Hz, 1H), 7.15 (ddd, $J = 7.7$, 4.9, 1.0 Hz, 1H), 3.84 (d, $J = 7.5$ Hz, 2H), 2.03 (t, $J = 7.5$ Hz, 1H) ppm; ^{13}C NMR (100 MHz, $CDCl_3$, 25 °C): $\delta = 160.2$,

149.4, 136.9, 122.3, 122.0, 30.9 ppm; HR ESI-TOF MS (H_2O, positive mode): *m*/*z* = 126.0370 (calculated *m*/*z* on the basis of the monoisotopic mass of C_6H_8NS $[M + H]^+$ =126.0377).

2) Synthesis of oPySS: To a degassed water solution (10 mL) of 2-(chloromethyl)pyridine (1.096 g, 6.682 mmol) was added thiourea (0.706 g, 9.27 mmol) under N_2, and the mixture was heated to 90 °C. After stirring for 1.5 h, to the mixture was added a degassed water (2 mL) solution of NaOH (1.012 g, 25.3 mmol). After stirring for additional 13 h at 90 °C, the reaction mixture was cooled to 25 °C. Then, to the mixture was added 3% H_2O_2 aq. (20 mL). After stirring for 1.5 h at 25 °C, the mixture was extracted with CH_2Cl_2 (20 mL, three times), and the organic extract was washed with brine (10 mL, once), dried over Na_2SO_4 and filtered off from insoluble substances through a filter paper. The filtrate was evaporated to dryness under reduced pressure, and the residue was chromatographed on silica gel with CH_2Cl_2 as an eluent to allow isolation of oPySS (0.343 g, 1.38 mmol) as reddish-brown oil in 41% yield.
TLC (CH_2Cl_2) R_f = 0.10; ^{1}H NMR (400 MHz, $CDCl_3$, 25 °C): δ = 8.59–8.57 (m, 2H), 7.65 (td, J = 7.8, 1.8 Hz, 2H), 7.26-7.18 (m, 4H), 3.85 (s, 4H) ppm; ^{13}C NMR (100 MHz, $CDCl_3$, 25 °C): δ = 157.2, 149.7, 136.5, 123.7, 122.3, 45.1 ppm; HR ESI-TOF MS (MeOH, positive mode): *m*/*z* = 249.0523 (calculated *m*/*z* on the basis of the monoisotopic mass of $C_{12}H_{13}N_2S_2$ $[M + H]^+$ = 249.0520).

3) Synthesis of oMePySS: To a dehydrated CH_3CN solution (1 mL) of oPySS (84.8 mg, 0.341 mmol) was added CH_3I (485 mg, 3.42 mmol) under N_2. After refluxing for 23 h, the reaction mixture was cooled to 25 °C followed by addition of CH_3CN (1 mL) and acetone (10 mL) for precipitation. Precipitates were collected by filtration and were dissolved in water followed by evaporation to dryness under reduced pressure to allow isolation of oMePySS (142 mg, 0.267 mmol) as pale-yellow solid in 78% yield.
^{1}H NMR (400 MHz, D_2O, 25 °C): δ = 8.66 (d, J = 6.0 Hz, 2H), 8.37 (td, J = 7.9, 1.1 Hz, 2H), 7.86–7.80 (m, 4H), 4.24 (s, 4H), 4.21 (s, 6H) ppm; ^{13}C NMR (100 MHz, D_2O, 25 °C): δ = 152.1, 147.5, 145.6, 130.3, 126.9, 45.7, 37.4 ppm; HR ESI-TOF MS (CH_3CN, positive mode): *m*/*z* = 139.0458 (calculated *m*/*z* on the basis of the monoisotopic mass of $C_{14}H_{18}N_2S_2$ $[M]^{2+}$ = 139.0450).

4) Synthesis of oMePySH: To a degassed water solution (1.0 mL) of oMePySS (39.0 mg, 0.0733 mmol) was added DTT (45.0 mg, 0.299 mmol) under N_2. After stirring for 18 h at 25 °C, the reaction mixture was chromatographed on preparative reversed-phase HPLC with a mixture of solvent A (water containing 0.1 v/v% CF_3CO_2H) and solvent B (CH_3CN containing 0.1 v/v% CF_3CO_2H) as an eluent under a linear gradient profile (flow rate: 18.9 mL min^{-1}, percentage of solvent A: 0% at 0 min, 96% at 10 min, 93% at 20 min). Collected fraction containing oMePySH was evaporated to dryness under reduced pressure, and to the residue was added water (1 mL) and 2 M HCl aq. (1 mL), and the mixture was stirred for 1 h at 25 °C under N_2 and evaporated under reduced pressure to allow isolation of oMePySH (25.2 mg, 0.143 mmol) as pale-yellow oil in 98% yield.

^{1}H NMR (400 MHz, D_2O, 25 °C): δ = 8.57 (d, J = 6.4 Hz, 1H), 8.30 (td, J = 7.9, 1.2 Hz, 1H), 7.93–7.91 (m, 1H), 7.75–7.71 (m, 1H), 4.21 (s, 3H), 4.03 (s, 2H) ppm; ^{13}C NMR (100 MHz, D_2O, 25 °C): δ = 156.5, 146.6, 146.2, 128.5, 126.2, 45.2, 24.8 ppm; HR ESI-TOF MS (H_2O, positive mode): m/z = 140.0529 (calculated m/z on the basis of the monoisotopic mass of $C_7H_{10}NS$ $[M]^+$ = 140.0534).

5) Synthesis of mPySH: To a degassed water solution (10 mL) of 3-(chloromethyl)pyridine (1.147 g, 6.990 mmol) was added thiourea (1.066 g, 14.00 mmol) under N_2, and the mixture was heated to 90 °C. After stirring for 2 h, to the mixture was added a degassed water (2 mL) solution of NaOH (0.845 g, 21.1 mmol). After stirring for additional 2 h at 90 °C, the reaction mixture was cooled to 25 °C. The mixture was washed out with *tert*-butyl methyl ether (10 mL, three times). The resulting aqueous mixture was neutralized to pH 7 with 1 M HCl aq. followed by extraction with CH_2Cl_2 (10 mL, three times). The organic extract was washed with brine (10 mL, once), dried over Na_2SO_4 and filtered off from insoluble substances through a filter paper. The filtrate was evaporated to dryness under reduced pressure, and the residue was chromatographed on silica gel with CH_2Cl_2 as an eluent to allow isolation of mPySH (0.656 g, 5.24 mmol) as colorless oil in 75% yield.

TLC (CH_2Cl_2) R_f = 0.28; ^{1}H NMR (400 MHz, $CDCl_3$, 25 °C): δ = 8.55 (d, J = 2.3 Hz, 1H), 8.49 (dd, J = 4.8, 1.6 Hz, 1H), 7.67 (dt, J = 7.8, 2.3 Hz, 1H), 7.27–7.23 (m, 1H), 3.74 (d, J = 7.6 Hz, 2H), 1.84 (t, J = 7.6 Hz, 1H) ppm; ^{13}C NMR (100 MHz, $CDCl_3$, 25 °C): δ = 149.3, 148.5, 136.7,

135.7, 123.6, 26.2 ppm HR ESI-TOF MS (H_2O, positive mode): *m*/*z* = 126.0372 (calculated *m*/*z* on the basis of the monoisotopic mass of C_6H_8NS $[M + H]^+$ = 126.0377).

6) Synthesis of mPySS: To an aqueous solution (4.1 mL) of mPySH (486 g, 3.88 mmol) was added 1 M NaOH aq. (435 μL) and 3% H_2O_2 (2.61 mL) at 25 °C. After stirring for 24 h, the mixture was extracted with CH_2Cl_2 (20 mL, three times), and the organic extract was washed with brine (20 mL, once), dried over Na_2SO_4 and filtered off from insoluble substances through a filter paper. The filtrate was evaporated to dryness under reduced pressure, and the residue was chromatographed on silica gel with CH_2Cl_2/MeOH (40/1 v/v) as an eluent to allow isolation of mPySS (0.432 g, 1.74 mmol) as pale-pink oil in 90% yield.

TLC (CH_2Cl_2/MeOH=40/1) R_f = 0.13; ^{1}H NMR (400 MHz, $CDCl_3$, 25 °C): δ = 8.54 (dd, J = 4.8, 1.6 Hz, 2H), 8.46 (d, J = 1.6 Hz, 2H), 7.57 (dt, J = 7.8, 2.1 Hz, 2H), 7.29–7.26 (m, 2H), 3.58 (s, 4H) ppm; ^{13}C NMR (100 MHz, $CDCl_3$, 25 °C): δ = 149.8, 148.1, 138.3, 134.9, 124.4, 39.0 ppm; HR ESI-TOF MS (MeOH, positive mode): *m*/*z* = 249.0520 (calculated *m*/*z* on the basis of the monoisotopic mass of $C_{12}H_{13}N_2S_2$ $[M + H]^+$ = 249.0520).

7) Synthesis of mMePySS: To a dehydrated CH_3CN solution (2 mL) of mPySS (108 mg, 0.435 mmol) was added CH_3I (622 mg, 4.38 mmol) under N_2. After stirring for 25 h at 60 °C, the reaction mixture was cooled to 25 °C followed by addition of acetone (10 mL) for precipitation. Precipitates were collected by filtration and were dissolved in water followed by evaporation to dryness under reduced pressure to allow isolation of mMePySS (194 mg, 0.364 mmol) as white solid in 84% yield.

^{1}H NMR (400 MHz, D_2O, 25 °C): δ = 8.68 (s, 2H), 8.57 (d, J = 6.4 Hz, 2H), 8.35 (d, J = 8.2 Hz, 2H), 7.88 (dd, J = 8.2, 6.4 Hz, 2H), 4.25 (s, 6H), 3.92 (s, 4H) ppm; ^{13}C NMR (100 MHz, D_2O, 25 °C): δ = 145.7, 144.9, 143.9, 139.3, 127.7, 48.3, 36.6 ppm; HR ESI-TOF MS (CH_3CN, positive mode): *m*/*z* = 139.0459 (calculated *m*/*z* on the basis of the monoisotopic mass of $C_{14}H_{18}N_2S_2$ M^{2+} = 139.0450).

8) Synthesis of mMePySH: To a degassed water solution (1.4 mL) of mMePySS (51.0 mg, 0.0958 mmol) was added DTT (118 mg, 0.785 mmol) under N_2. After stirring for 14 h at 25 °C, the reaction mixture was chromatographed on preparative reversed-phase HPLC with a mixture

of solvent A (water containing 0.1 v/v% CF_3CO_2H) and solvent B (CH_3CN containing 0.1 v/v% CF_3CO_2H) as an eluent under a linear gradient profile (flow rate: 18.9 mL min^{-1}, percentage of solvent A: 0% at 0 min, 96% at 10 min, 93% at 20 min). Collected fraction containing mMePySH was evaporated to dryness under reduced pressure, and to the residue was added water (1 mL) and 2 M HCl aq. (1 mL), and the mixture was stirred for 1 h at 25 °C under N_2 and evaporated under reduced pressure to allow isolation of mMePySH (18.0 mg, 0.102 mmol) as colorless oil in 53% yield.

^{1}H NMR (400 MHz, D_2O, 25 °C): δ = 8.67 (s, 1H), 8.50 (d, J = 6.2 Hz, 1H), 8.36 (d, J = 7.8 Hz, 1H), 7.82 (dd, J = 7.8, 6.2 Hz, 1H), 4.21 (s, 3H), 3.80 (s, 2H) ppm; ^{13}C NMR (100 MHz, D_2O, 25 °C): δ = 144.8, 144.5, 143.3, 143.0, 127.7, 48.0, 24.4 ppm; HR ESI-TOF MS (H_2O, positive mode): m/z = 140.0531 (calculated m/z on the basis of the monoisotopic mass of $C_7H_{10}NS$ M^+ = 140.0534).

9) Synthesis of pPySH: To a degassed water solution (10 mL) of 4-(chloromethyl)pyridine (1.150 g, 7.008 mmol) was added thiourea (1.067 g, 14.02 mmol) under N_2, and the mixture was heated to 90 °C. After stirring for 2 h, to the mixture was added a degassed water (2 mL) solution of NaOH (0.847 g, 21.2 mmol). After stirring for additional 2 h at 90 °C, the reaction mixture was cooled to 25 °C. The mixture was washed out with *tert*-butyl methyl ether (10 mL, three times). The resulting aqueous mixture was neutralized to pH 7 with 1 M HCl aq. followed by extraction with CH_2Cl_2 (10 mL, three times). The organic extract was washed with brine (10 mL, once), dried over Na_2SO_4 and filtered off from insoluble substances through a filter paper. The filtrate was evaporated to dryness under reduced pressure, and the residue was chromatographed on silica gel with CH_2Cl_2 as an eluent to allow isolation of pPySH (0.374 g, 2.98 mmol) as colorless oil in 43% yield.

TLC (CH_2Cl_2) R_f = 0.25; ^{1}H NMR (400 MHz, $CDCl_3$, 25 °C): δ = 8.54 (q, J = 2.0 Hz, 2H), 7.25 (q, J = 2.0 Hz, 2H), 3.69 (d, J = 7.8 Hz, 2H), 1.84 (t, J = 7.8 Hz, 1H) ppm; ^{13}C NMR (100 MHz, $CDCl_3$, 25 °C): δ = 150.1, 149.8, 123.1, 27.9 ppm; HR ESI-TOF MS (H_2O, positive mode): m/z = 126.0373 (calculated m/z on the basis of the monoisotopic mass of C_6H_8NS $[M + H]^+$ =126.0377).

10) Synthesis of pPySS: To an aqueous solution (3.1 mL) of pPySH (0.367 g, 2.93 mmol) was added 1 M NaOH aq. (660 μL) and 3% H_2O_2 (1.98 mL) at 25 °C. After stirring for 24 h, the mixture was extracted with CH_2Cl_2 (20 mL, three times), and the organic extract was washed with brine (20 mL, once), dried over Na_2SO_4 and filtered off from insoluble substances through a filter paper. The filtrate was evaporated to dryness under reduced pressure, and the residue was chromatographed on silica gel with CH_2Cl_2/MeOH (20/1 v/v) as an eluent to allow isolation of pPySS (0.262 g, 1.05 mmol) as pale-yellow solid in 72% yield.

TLC (CH_2Cl_2/MeOH=20/1) $R_f = 0.30$; ^{1}H NMR (400 MHz, $CDCl_3$, 25 °C): $\delta = 8.57$ (q, $J = 2.0$ Hz, 4H), 7.16 (q, $J = 2.0$ Hz, 4H), 3.58 (s, 4H) ppm; ^{13}C NMR (100 MHz, CD_3OD, 25 °C): $\delta =$ 150.2, 149.6, 126.2, 41.8 ppm; HR ESI-TOF MS (MeOH, positive mode): m/z = 249.0525 (calculated m/z on the basis of the monoisotopic mass of $C_{12}H_{13}N_2S_2$ $[M + H]^+$ = 249.0520).

11) Synthesis of pMePySS: To a dehydrated CH_3CN solution (2 mL) of pPySS (92.5 mg, 0.372 mmol) was added CH_3I (541 mg, 3.81 mmol) under N_2. After refluxing for 23 h at 90 °C, the reaction mixture was cooled to 25 °C followed by addition of acetone (10 mL) for precipitation. Precipitates were collected by filtration and were dissolved in water followed by evaporation to dryness under reduced pressure to allow isolation of pMePySS (166 mg, 0.312 mmol) as yellowish blown solid in 84% yield.

^{1}H NMR (400 MHz, D_2O, 25 °C): $\delta = 8.62$ (d, $J = 6.9$ Hz, 4H), 7.89 (d, $J = 6.9$ Hz, 4H), 4.25 (s, 6H), 3.99 (s, 4H) ppm; ^{13}C NMR (100 MHz, D_2O, 25 °C): $\delta = 157.5$, 144.9, 128.3, 48.0, 39.6 ppm; HR ESI-TOF MS (CH_3CN, positive mode): m/z = 139.0455 (calculated m/z on the basis of the monoisotopic mass of $C_{14}H_{18}N_2S_2$ M^{2+} = 139.0450).

12) Synthesis of pMePySH: To a degassed water solution (1.0 mL) of pMePySS (37.9 mg, 0.0712 mmol) was added DTT (44.3 mg, 0.295 mmol) under N_2. After stirring for 17 h at 25 °C, the reaction mixture was chromatographed on preparative reversed-phase HPLC with a mixture of solvent A (water containing 0.1 v/v% CF_3CO_2H) and solvent B (CH_3CN containing 0.1 v/v% CF_3CO_2H) as an eluent under a linear gradient profile (flow rate: 18.9 mL min^{-1}, percentage of solvent A: 0% at 0 min, 96% at 10 min, 93% at 20 min). Collected fraction containing pMePySH was evaporated to dryness under reduced pressure, and to the residue was added water (1 mL) and 2 M HCl aq. (1 mL), and the mixture was stirred for 1 h at 25 °C under N_2

and evaporated under reduced pressure to allow isolation of pMePySH (21.6 mg, 0.123 mmol) as white solid in 86% yield.

^{1}H NMR (400 MHz, D_2O, 25 °C): δ = 8.51 (d, J = 6.9 Hz, 2H), 7.85 (d, J = 6.9 Hz, 2H), 4.18 (s, 3H), 3.82 (s, 2H) ppm; ^{13}C NMR (100 MHz, D_2O, 25 °C): δ = 161.5, 144.8, 127.2, 47.5, 26.9 ppm; HR ESI-TOF MS (H_2O, positive mode): m/z = 140.0531 (calculated m/z on the basis of the monoisotopic mass of $C_7H_{10}NS$ M^+ = 140.0534).

5. HPLC analysis of a mixture of pMePySH and GSSG

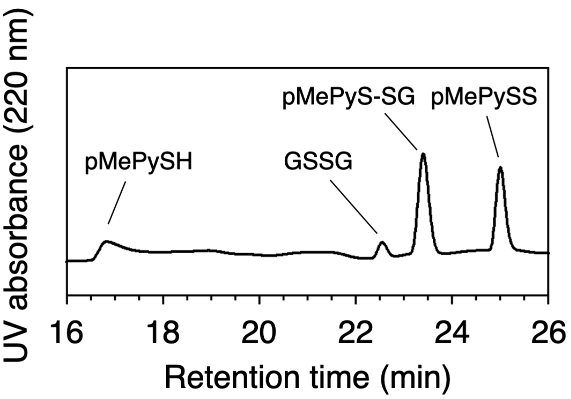

Fig. S3 HPLC analysis of a mixture of pMePySH (1.0 mM) and GSSG (0.2 mM) in a buffer (50 mM Tris-HCl, 300 mM NaCl, pH 7.5) after 10 min incubation at 30 ºC. The mixture (30 µL) was added to 1 M HCl aq. (70 µL), then the sample solution was analyzed by HPLC immediately at a flow rate of 1.0 mL min^{-1} monitoring at 220 nm with a linear gradient of elution (solvent A: water containing 0.1% trifluoroacetic acid; solvent B: acetonitrile containing 0.1% trifluoroacetic acid; percentages of solvent B: 0% during 0–8 min, 6% at 15 min, 10% at 30 min). YMC Triart C18 column (φ4.6 × 250 mm) was used for the analysis.

6. HPLC analysis of BPTI folding

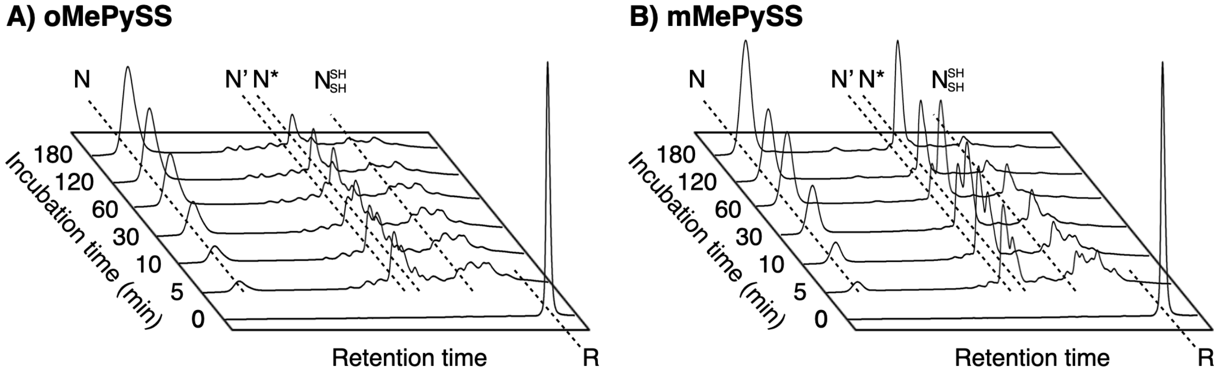

Fig. S4 Time-course reverse-phase HPLC analyses of oxidative folding of BPTI (30 μM) in the presence of (A) oMePySS (90 μM) and (B) mMePySS (90 μM) between 16- and 41-min retention time. N and R depict native and reduced forms of BPTI, respectively. Eluent buffers: water (containing 0.05% TFA) and CH_3CN (containing 0.05% TFA) with a linear gradient; flow rate: 1.0 mL min^{-1}; detection wavelength: 229 nm; temperature: 30 °C.

7. Enzymatic activity recovery of RNase A

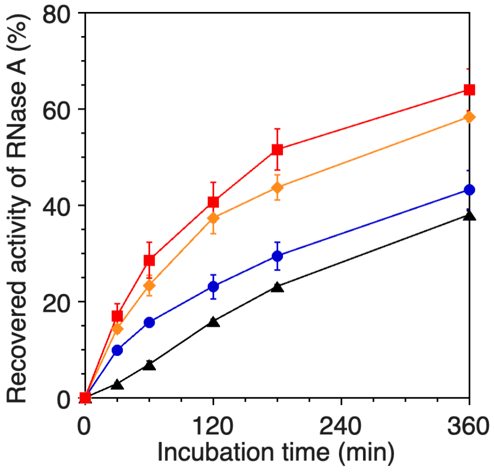

Fig. S5 Time-course plots of recovered enzymatic activity of RNase A (40 µM) in the presence of GSSG (160 µM) and (blue circles) oMePySH, (orange diamonds) mMePySH, (red squares) pMePySH, and (black triangles) GSH (320 µM) in a buffer (50 mM Tris-HCl, 300 mM NaCl, pH 7.5) at 30 °C. The activity was evaluated by spectroscopic monitoring of the hydrolysis of cCMP to 3'-CMP at 30 °C. Error bars indicate the means ± SEM of three independent experiments.

8. HPLC analysis of insulin folding

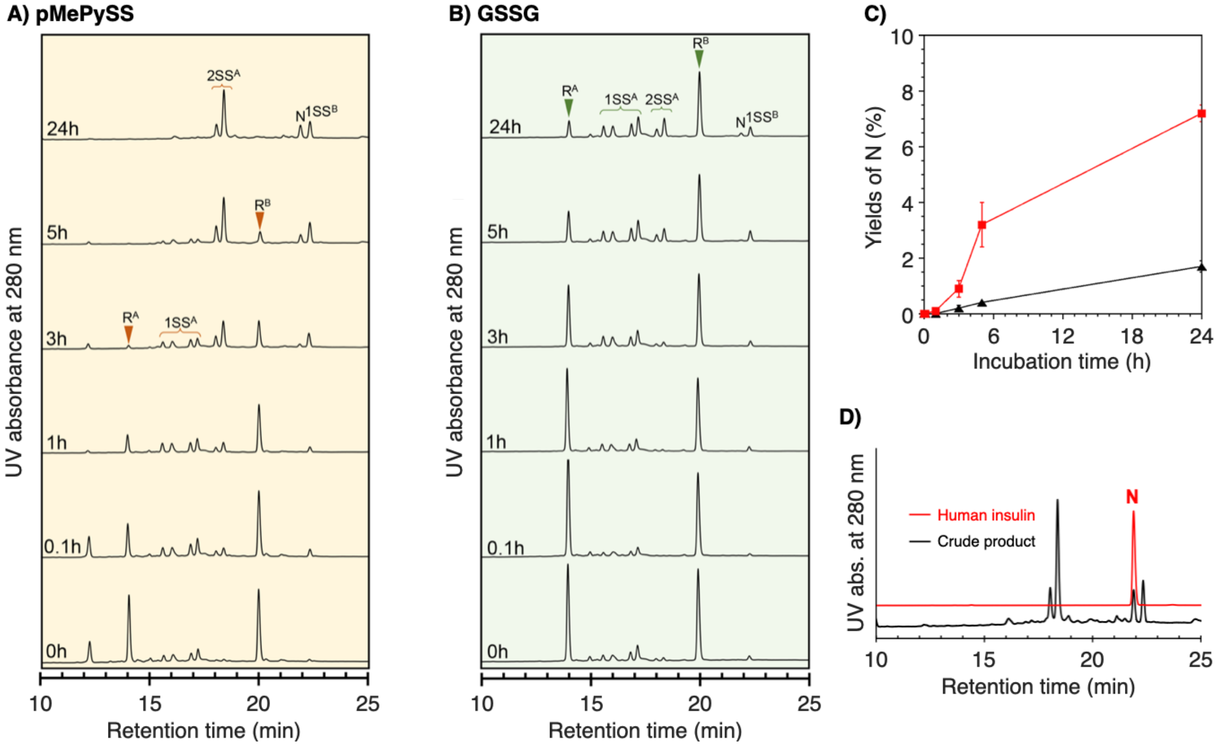

Fig. S6 Time-course reverse-phase HPLC analyses of oxidative folding of human insulin by mixing R^A (200 μM, A-chain), R^B (200 μM, B-chain) and A) pMePySS (200 μM) or B) GSSG (200 μM). C) Time-course plots of the yields of native insulin in the presence of (red squares) pMePySS and (black triangles) GSSG. Error bars indicate the means ± SEM of three independent experiments. D) HPLC traces of human insulin (red, standard sample) and crude product obtained by mixing R^A, R^B, and pMePySS (black). This comparison indicates that the fraction of the crude product at the retention time of 21.9 min corresponds to the native form of human insulin.

The oxidative folding was performed at pH 10.0 and –10 °C in the presence of 0.48 M urea. Before HPLC analyses, aliquots of the folding solution were pre-treated with aqueous 2-aminoethyl methanethiosulfonate (AEMTS) to quench the reaction by blocking the free SH groups into $-SSCH_2CH_2NH_3^+$. The detailed folding pathway of insulin is described in a previous paper.[2]

R^A: reduced form of chain A, R^B: reduced form of chain B, $1SS^A$: folding intermediates of insulin chain A with one intra-SS bond, $2SS^A$: folding intermediates of insulin chain A with

two intra-SS bonds, $1SS^B$: folding intermediates of insulin chain B with one intra-SS bond, N: the native form of human insulin.

9. ELISA analysis of insulin

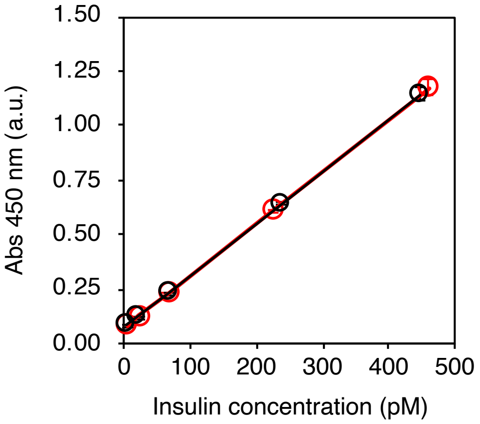

Fig. S7 ELISA analysis of human insulin (black, standard sample) and human insulin obtained by the oxidative two-chain folding using R^A, R^B, and pMePySS and subsequent HPLC purification (red). Error bars indicate the means ± SEM of three independent experiments. The lyophilized insulin was dissolved in 10 mM HCl and diluted to respective concentrations in 50 mM HEPES-NaOH buffer (pH 7.5). All samples were analyzed by the human insulin ELISA kit (Mercodia). The turbidity at 450 nm was measured using a Varioskan (Thermo Fisher Scientific).

10. Supplementary references

1. T. W. Bruice and G. L. Kenyon, *J. Protein Chem.*, 1982, **1**, 47–58.
2. K. Arai, T. Takei, R. Shinozaki, M. Noguchi, S. Fujisawa, H. Katayama, L. Moroder, S. Ando, M. Okumura, K. Inaba, H. Hojo and M. Iwaoka, *Commun. Chem.*, 2018, **1**, 26.
3. G. L. Ellman, *Arch. Biochem. Biophys.*, 1959, **82**, 70–77.
4. M. M. Lyles and H. F. Gilbert, *Biochemistry*, 1991, **30**, 613–619.
5. U. K. Laemmli, *Nature*, 1970, **227**, 680–685.
6. K. Arai, H. Ueno, Y. Asano, G. Chakrabarty, S. Shimodaira, G. Mugesh and M. Iwaoka, *ChemBioChem*, 2018, **19**, 207–211.
